# Spontaneous Atraumatic Mediastinal Hemorrhage: Challenging Management of a Life-Threatening Condition and Literature Review

**DOI:** 10.1177/2324709613484451

**Published:** 2013-04-01

**Authors:** Morkos Iskander, Khurram Siddique, Anil Kaul

**Affiliations:** 1 St Helen’s & Knowsley NHS Trust, Whiston Hospital, Liverpool, UK

**Keywords:** spontaneous, hematoma, mediastinum

## Abstract

Spontaneous atraumatic mediastinal hematomas are rare. We present a case of a previously fit and well middle-aged lady who presented with acute breathlessness and an increasing neck swelling and spontaneous neck bruising. On plain chest radiograph, widening of the mediastinum was noted. The bruising was later confirmed to be secondary to mediastinal hematoma. This life-threatening diagnostic conundrum was managed conservatively with a multidisciplinary team approach involving upper gastrointestinal and thoracic surgeons, gastroenterologists, radiologists, intensivists, and hematologists along with a variety of diagnostic modalities. A review of literature is also presented to help surgeons manage such challenging and complicated cases.

## Case Report

**Figure 1.**
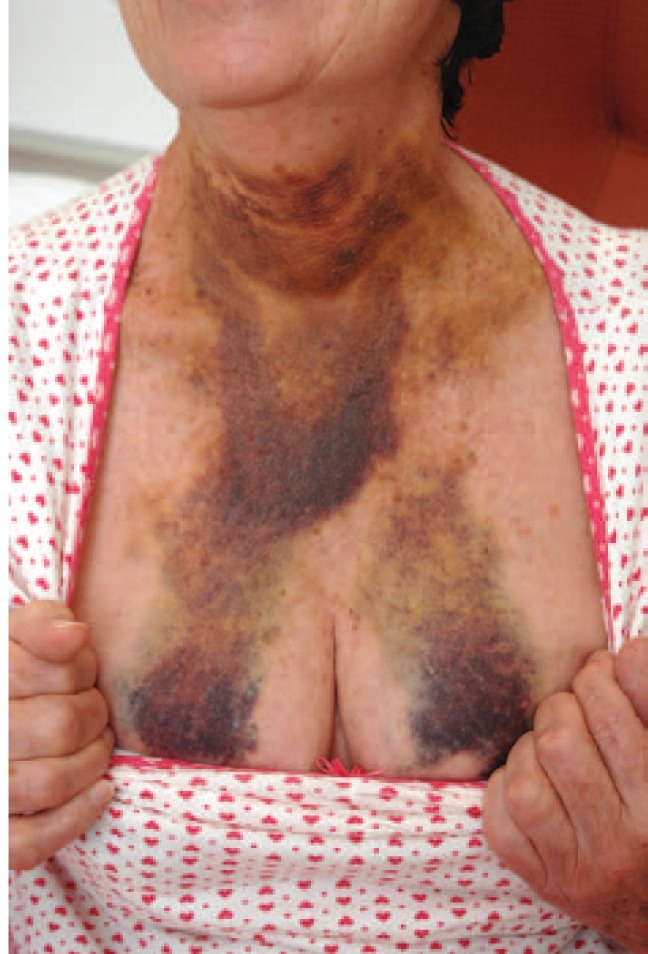
A photograph demonstrating the extent of bruising at presentation.

A 59-year-old woman presented to the emergency department with a 1-day history of acute chest tightness with exertional dyspnea, an increasing neck swelling, and spontaneous neck bruising (Figure 1). The neck swelling has increased gradually over 2 days, leading to intermittent dysphagia. She denied any history of trauma, vomiting, regurgitation of food, bouts of cough, palpitations, or any other constitutional symptoms. Her past history included a history of paroxysmal atrial fibrillation and gastroesophageal reflux disease. There was no history of bleeding disorders. At presentation she was pale and sweaty. Her vitals were pulse 87 beats/min, blood pressure of 143/57 mm Hg, respiratory rate 16, and oxygen saturation 96% on room air.

Chest examination revealed extensive bruising extending along both sides of her neck, over the sternum to the upper breasts. A firm neck swelling over the trachea was also palpable. Auscultation confirmed bibasal crepitations. No palpable lymph nodes or any other swelling was noted in her cervical region. Her blood results showed hemoglobin of 12.7 gm/dL, white cell count of 6.8 × 10^9^/L, and platelets of 217 × 10^9^/L. Coagulation screen showed prothrombin time of 10.6, activated partial thromboplastin time 24, and international normalized ratio of 1.0.

An initial plain chest radiograph demonstrated a prominent superior mediastinum.

**Figure 2.**
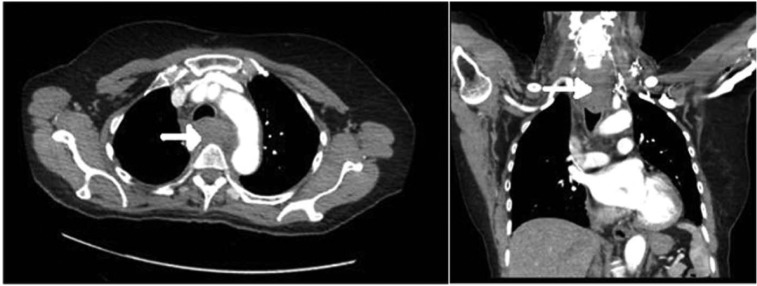
CT thorax with contrast at presentation. The hematoma can be seen causing mediastinal compression.

An urgent contrast-enhanced computed tomography (CT) scan of her thorax and neck was performed. The CT showed a large prevertebral soft tissue mass extending from the level of the larynx down to below the carina to about the level of T8 vertebral body (Figure 2). The soft tissue mass extended superolaterally to the borders of the carotid sheath and also pushed esophagus anteriorly to bulge into the back of the trachea.

Free air along with a fluid level was noted within esophageal mass and posterior mediastinum at the level of the tracheal bifurcation, suggestive of esophageal perforation. A water-soluble contrast swallow was subsequently performed, which did not show esophageal perforation though the upper esophagus was distorted due to soft tissue swelling. An urgent gastroscopy was also performed, which showed a normal esophagus without any intramural mass, ulcers, or growth and normal stomach and duodenum.

The case was discussed at the multidisciplinary team meeting, involving upper gastrointestinal surgeons, gastroenterologists, radiologists, intensivists, hematologists, and specialist nurses, since it represented a diagnostic conundrum. After reviewing the case, a conservatively approach was adopted, and the patient was admitted for observation and managed on a surgical ward. The patient remained hemodynamically stable from day 1 to day 4 without any deterioration of her clinical condition or any drop in her hemoglobin level. A repeat CT scan thorax was performed on the fifth postadmission day, which showed a persistent swelling in the mediastinum that had slightly reduced in size.

Extensive blood investigations were performed, including von Willebrand factor, antiphospholipid syndrome antibody, myeloma screening, hepatitis profile, and thyroid function tests, as per advice of hematologists but no abnormality was identified.

**Figure 3.**
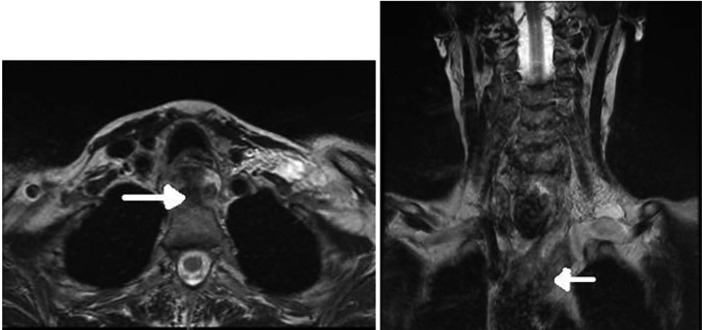
MRI neck with contrast at day 7. Residual hematoma is seen in the superior mediastinum and extending into the neck along fascial layers.

The case was discussed at the specialist upper gastrointestinal multidisciplinary team meeting as well, with a recommendation for further cross-sectional imaging. A magnetic resonance (MR) scan of the neck and endoscopic ultrasound were performed. The MR confirmed a mediastinal hematoma (MH; Figure 3), and endoscopic ultrasound did not show any esophageal pathology.

The patient was discharged home after 10 days and remained stable throughout her stay. Her neck and chest wall bruising had started to disappear. All her symptoms completely resolved within 8 weeks. She has been enjoying a good quality of life after discharge and was kept on a regular follow-up.

## Discussion

The review of literature identifies various causes of acute mediastinal hemorrhage, majority of which are life threatening. These include major thoracic trauma,^1^ cardiac and great vessel aneurysm, dissection or rupture,^2^ hypertension,^3^ or iatrogenic causes including invasive procedures,^4^ as well as valsalva maneuver^5^ and iatrogenic causes. However, spontaneous mediastinal hematomas are a rare but very important presentation, which if not dealt with caution can lead to rapid deterioration and death. Shimokawa et al^6^ have mentioned the 4 clinical settings where spontaneous MH can occur: (*a*) as a complication of enlarging mediastinal masses, (*b*) sustained hypertension, (*c*) hematological conditions, and (*d*) transient increase in intrathoracic pressure.

A high index of suspicion for MH should be kept for any patient who presents with symptoms of acute superior mediastinal compression, chest radiograph showing widened mediastinum, and bruising over the neck and upper chest. Urgent imaging should be performed to confirm the diagnosis. Angiography and surgical exploration used to be the gold standard for diagnosis, but they have been replaced by contrast-enhanced spiral CT of the chest, magnetic resonance imaging scan, or positron emission tomography.^7^ It has been shown in the literature that the sensitivity and specificity of combined modalities to detect MH is extremely high.^2,7^ Hence, in our case we did use various diagnostic tools to help find the underlying cause. We recommend a multidisciplinary team approach to manage such challenging cases since there is a possibility of rapidly changing scenario as well as multiple aspects of care that need addressing at the same time. However, the treating clinician must bear in mind that sometimes the exact source of the hemorrhage remains unknown despite careful radiological investigations and even thoracotomy.^8^

The initial management is fluid resuscitation, transfusion if required, and urgent imaging.^9^ Endotracheal intubation is performed for airway compromise.^2,9^ Based on the patient’s condition and the diagnostic findings, the treatment options include the following: (*a*) operative intervention (cardiorespiratory compromise), (*b*) angiography and embolization, or (*c*) careful watch and wait (for stable patient) as in our patient.^5^

Although in our patient a very large hematoma was seen, she was successfully managed conservatively without any intervention and her symptoms improved gradually.

## Conclusions

Spontaneous mediastinal hematomas are rare. A multidisciplinary team approach should be used to manage these patients conservatively. The exact source of the hemorrhage may remain unknown despite careful radiological investigations and even thoracotomy.
